# Induction of Heme Oxygenase-1 with Hemin Reduces Obesity-Induced Adipose Tissue Inflammation via Adipose Macrophage Phenotype Switching

**DOI:** 10.1155/2014/290708

**Published:** 2014-11-11

**Authors:** Thai Hien Tu, Yeonsoo Joe, Hye-Seon Choi, Hun Taeg Chung, Rina Yu

**Affiliations:** ^1^Department of Food Science and Nutrition, University of Ulsan, Ulsan 680-749, Republic of Korea; ^2^Department of Biological Science, University of Ulsan, Ulsan 680-749, Republic of Korea

## Abstract

Adipose macrophages with the anti-inflammatory M2 phenotype protect against obesity-induced inflammation and insulin resistance. Heme oxygenase-1 (HO-1), which elicits antioxidant and anti-inflammatory activity, modulates macrophage phenotypes and thus is implicated in various inflammatory diseases. Here, we demonstrate that the HO-1 inducer, hemin, protects against obesity-induced adipose inflammation by inducing macrophages to switch to the M2 phenotype. HO-1 induction by hemin reduced the production of proinflammatory cytokines (TNF-*α* and IL-6) from cocultured adipocytes and macrophages by inhibiting the activation of inflammatory signaling molecules (JNK and NF-*κ*B) in both cell types. Hemin enhanced transcript levels of M2 macrophage marker genes (IL-4, Mrc1, and Clec10a) in the cocultures, while reducing transcripts of M1 macrophage markers (CD274 and TNF-*α*). The protective effects of hemin on adipose inflammation and macrophage phenotype switching were confirmed in mice fed a high-fat diet, and these were associated with PPAR*γ* upregulation and STAT6 activation. These findings suggest that induction of HO-1 with hemin protects against obesity-induced adipose inflammation through M2 macrophage phenotype switching, which is induced by the PPAR*γ* and STAT6 pathway. HO-1 inducers such as hemin may be useful for preventing obesity-induced adipose inflammation.

## 1. Introduction

Obesity-induced adipose inflammation plays an important role in the development of metabolic complications such as insulin resistance and type 2 diabetes [1–3]. The accumulation of adipose tissue macrophages (ATMs) is a hallmark of obesity-induced adipose inflammation, and inflammatory mediators (TNF-*α*, IL-6, and MCP-1) released from the ATMs play a crucial role in promoting obesity-related systemic inflammatory conditions [4]. Interestingly, ATMs can polarize into different activation states that perform different functions by producing proinflammatory or anti-inflammatory cytokines [4], depending on microenvironmental stimuli. Proinflammatory macrophages (M1) are classically activated by interferon-*γ* or lipopolysaccharide [5, 6], while anti-inflammatory macrophages (M2) are activated by IL-4 or IL-13 [4–6]. The ATMs in lean mice have an M2 profile, whereas those in obese mice are polarized towards the M1 phenotype [4]. This suggests that agents that polarize macrophages towards the M2 phenotype might protect against obesity-induced adipose inflammation.

Heme oxygenase-1 (HO-1) is a microsomal enzyme induced in response to oxidative stress and inflammatory stimuli, which plays an important role in suppressing inflammation and insulin resistance [7]. It catalyzes the oxidative degradation of heme to biliverdin and carbon monoxide (CO) [8], and its enzymatic activity is paralleled by the levels of its transcripts and protein [8, 9]. Importantly, the induction of HO-1 has potent anti-inflammatory effects against macrophage-mediated inflammatory responses by preferentially promoting the M2 phenotype [9, 10]. Moreover, induction of HO-1 in genetically obese mice (ob/ob) and diabetic rats increases adiponectin expression and suppresses inflammatory cytokine expression [11, 12]. However it remains unclear whether HO-1 induction reduces obesity-induced adipose inflammation by influencing adipose macrophage polarization.

Here, we demonstrate that HO-1 induction by hemin reduces levels of inflammatory cytokines and enhances adipose macrophage switching toward the M2 phenotype* in vitro* and* in vivo*. The HO-1 inducer hemin may be beneficial for protecting obesity-induced adipose tissue inflammation.

## 2. Materials and Methods

### 2.1. Reagents

Tricarbonyldichlororuthenium(II) dimer [Ru(CO)_3_Cl_2_]_2_ (Sigma-Aldrich, St. Louis, MO) known as CO-releasing molecule (CORM-2) was used as carbon monoxide (CO) donor. Because CORM-2 contained ruthenium (Ru) as their metal center, ruthenium(III) chloride hydrate (RuCl_3_) (Sigma-Aldrich, St. Louis, MO) was used as negative control. RuCl_3_ has the same basic structure as CORM-2 with the notable exception that it does not yield CO in solution [13]. CORM-2 or RuCl_3_ was solubilized in dimethyl sulfoxide (DMSO) to give a stock concentration of 1 M. Hemin, an inducer of HO-1 expression and activity which increases endogenously generated CO, and protoporphyrin IX zinc(II) (ZnPP), an inhibitor of HO-1 activity, were also purchased from Sigma-Aldrich (St. Louis, MO) and dissolved in 20 mM sodium hydroxide (NaOH) to give a stock concentration of 1 mM.

### 2.2. Animals

Six-week-old male C57BL/6 mice were purchased from Orient Ltd. (Busan, Korea). The mice were maintained under a standard light cycle (12 h light/dark) and were allowed free access to water and food. They were randomly assigned to the following experimental groups (*n* = 5 per group): (1) control diet + vehicle, (2) control diet + hemin, (3) high-fat diet (HFD) + vehicle, (4) HFD + hemin, and (5) HFD + hemin + ZnPP. The control diet contained 10% of its calories as fat while the HFD contained 60% of its calories as fat from lard and soybean oil (Research Diets Inc., New Brunswick, NJ); hemin and ZnPP (Sigma-Aldrich) were dissolved in 10% ammonium hydroxide (NH_4_OH) in 0.15 M NaCl as a stock solution of 100 mg/mL and then further diluted 1 : 40 with sterile 0.15 M NaCl. Hemin was intraperitoneally injected alone (25 mg/kg BW) or in combination with ZnPP (12.5 mg/kg BW) into the mice three times per week for 2 weeks [14]. Vehicle-injected mice received an identical NH_4_OH-containing solution lacking hemin or ZnPP. All animal experiments were approved by the animal ethics committee of the University of Ulsan and conformed to National Institutes of Health guidelines. Mice were killed after a 4 h fast, and blood was collected by heart puncture.

### 2.3. Cell Cultures and Treatments

Cells of the murine macrophage cell line Raw264.7 were obtained from the Korean Cell Line Bank (KCLB40071, Seoul, Korea), maintained in RPMI1640 (Gibco BRL, NY, USA) containing 10% (vol/vol) FBS (fetal bovine serum) (Gibco BRL, NY, USA) and incubated at 37°C in humidified 5% CO_2_. 3T3-L1 preadipocytes were grown in DMEM (Dulbecco's modified Eagle's medium) high glucose (Gibco BRL, NY, USA) containing 10% FBS. Differentiation of 3T3-L1 preadipocytes to mature adipocytes was induced with insulin, dexamethasone, and 3-isobutyl-1-methyl-xanthine, as described [15], and the differentiated 3T3-L1 cells were used on day 6 of differentiation. Coculture of adipocytes and macrophages was performed in a contact system: 3T3-L1 adipocytes (3 × 10^5^ cells/well) were incubated in 24-well plates and Raw264.7 macrophages (3 × 10^5^ cells/well) were placed onto the adipocytes. The adipocytes and macrophages were pretreated with hemin, ZnPP or CORM-2, and RuCl_3_ at the indicated concentrations for 1 h prior to coculture for 24 h. As a control, numbers of adipocytes and macrophages equal to those in the contact system were cultured separately and mixed after harvesting.

### 2.4. Separation of Adipocytes and Macrophages

Cocultures of equal numbers of 3T3-L1 adipocytes and Raw264.7 macrophages (as described earlier) were separated using the CD11b microbeads system (MACS; Miltenyi Biotec, Sunnyvale, CA, USA) according to the manufacturer's protocol. Briefly, cocultured cells were collected, washed twice with buffer (phosphate buffer saline (PBS) supplemented with 2 mM EDTA and 0.5% bovine serum albumin (BSA)), and incubated with CD11b microbeads for 15 min at 4°C. Washed and resuspended cells were applied to a MACS column, which retained CD11b^+^ cells and allowed negative cells (adipocytes) to pass through. The column was then removed from the separator and placed on a suitable collection tube. Appropriate amounts of column buffer were pipetted onto the column to flush out the positive cells (macrophages) using a plunger supplier with the column. This method resulted in 90% to 95% pure CD11b^+^ cells, as evaluated by flow cytometry.

### 2.5. Preparation of Adipocyte/Macrophage-Conditioned Medium

Adipocyte-conditioned medium was collected from 6-day matured 3T3-L1 adipocytes which were cultured in serum-free medium for 24 h. To prepare Raw264.7 macrophage-conditioned medium, macrophages were incubated for 24 h with 5 *μ*g/mL lipopolysaccharide (Sigma, St. Louis, MO, USA), washed once with PBS, and cultured in serum-free medium for another 24 h. These conditioned media were collected and filtered to remove debris.

### 2.6. Measurement of Cytokine Levels

Cytokine levels in culture supernatants were measured by enzyme-linked immunosorbent assays (ELISA) using an OptEIA mouse TNF-*α* set (BD Bioscience Pharmingen, CA, USA), a mouse IL-6, adiponectin set, and an IL-4 kit (R&D Systems, Minneapolis, MN). Values for cytokine levels were derived from standard curves using the curve-fitting program SOFTmax (Molecular Devices, Sunnyvale, CA, USA).

### 2.7. Quantitative Real-Time PCR (qRT-PCR)

Total RNA extracted from cultured cells was reverse-transcribed to generate cDNA using M-MLV reverse transcriptase (Promega, Madison, WI). Real-time PCR amplification of the cDNA was performed in duplicate with a SYBR premix Ex Taq kit (TaKaRa Bio Inc., Foster, CA) using a Thermal Cycler Dice (TaKaRa Bio Inc., Japan). All reactions were performed by the same procedure: initial denaturation at 95°C for 10 s, followed by 45 cycles of 95°C for 5 s and 60°C for 30 s. Results were analyzed with real-time system TP800 software, and all values for genes of interest were normalized to values for housekeeping genes (36B4 for cocultured cells; *β*-actin for adipose tissue). The mouse primer sequences were used as follows: adiponectin, 5′-GTCAGTGGATCTGACGACACCAA-3′ (Forward), 5′-ATGCCTGCCATCCAACCTG-3′ (Reverse); IL-4, 5′-ACGGAGATGGATGTGCCAAAC-3′ (Forward), 5′-AGCACCTTGGAAGCCCTACAGA-3′ (Reverse); CD274, 5′-GCCTCACTTGCTCATTACAGGTTC-3′ (Forward), 5′-GCAGTAGCTGTCAAGGGCTCA-3′ (Reverse); NOS2, 5′-CAAGCTGAACTTGAGCGAGGA-3′ (Forward), 5′-TTTACTCAGTGCCAGAAGCTGGA-3′ (Reverse); Mrc1, 5′-AGCTTCATCTTCGGGCCTTTG-3′ (Forward), 5′-GGTGACCACTCCTGCTGCTTTAG-3′ (Reverse); Clec10a, 5′-GGTCGTCTCCGTGATTGGAT-3′ (Forward), 5′-GGTGGTGTTGTCTAAAGTGGCTCTC-3′ (Reverse); HO-1, 5′-TGCAGGTGATGCTGACAGAGG-3′ (Forward), 5′-GGGATGAGCTAGTGCTGATCTGG-3′ (Reverse); TNF-*α*; IL-6; 36B4; *β*-actin [15].

### 2.8. Western Blot Analysis

The nuclear and cytosolic protein extracts were prepared using NE-PER Nuclear and Cytoplasmic Extraction Reagents (Thermo Scientific, Rockford, USA) according to the manufacturer's instruction. Epididymal adipose tissues were collected, washed in PBS, and homogenized in ice-cold CER I buffer. After incubation on ice for 10 min, ice-cold CER II was added to the cell suspension, mixed, and incubated for 1 min. The cytosolic extracts were collected after cells were centrifuged at 15,000 g for 5 min. The nuclear pellets were then resuspended in ice-cold NER and incubated for 40 min with vortexing for 15 s every 10 min. The nuclear extracts were collected after centrifugation (15,000 g for 10 min, 4°C). Other samples were lysed in lysis buffer (10 mM Tris-HCl, 10 mM NaCl, 0.1 mM EDTA, 50 mM NaF, 10 mM Na_4_P_2_O_7_, 1 mM MgCl_2_, 0.5% deoxycholate, 1% IGEPAL CA-630, and protease inhibitors cocktail) and centrifuged. The protein content of samples was determined using a BCA protein kit (Pierce, Rockford IL, USA). Samples containing 10–30 *μ*g of total protein were subjected to western blot analysis using polyclonal antibodies to phosphorylated JNK (p-JNK) (c-Jun amino-terminal kinase), total JNK, pSTAT6 (Tyr641), total STAT6, and histone H3 (Cell Signaling, Danvers, MA, USA); CD68, PPAR*γ*, I*κ*B*α* (inhibitor of nuclear factor-*κ*B alpha), and NF-*κ*B p65 (Santa Cruz Biotechnology, Santa Cruz, CA, USA); HO-1 (Enzo Life Sciences, Inc., Farmingdale, NY); and *β*-actin (Sigma).

### 2.9. Statistical Analysis

Results are presented as means ± SEM. Statistical comparisons were performed using Student's* t*-test with Duncan's multiple-range test. Differences were considered to be significant at *P* < 0.05.

## 3. Results

### 3.1. Hemin Induces HO-1 Expression in Macrophages and/or Adipocytes

**Figure 1:**
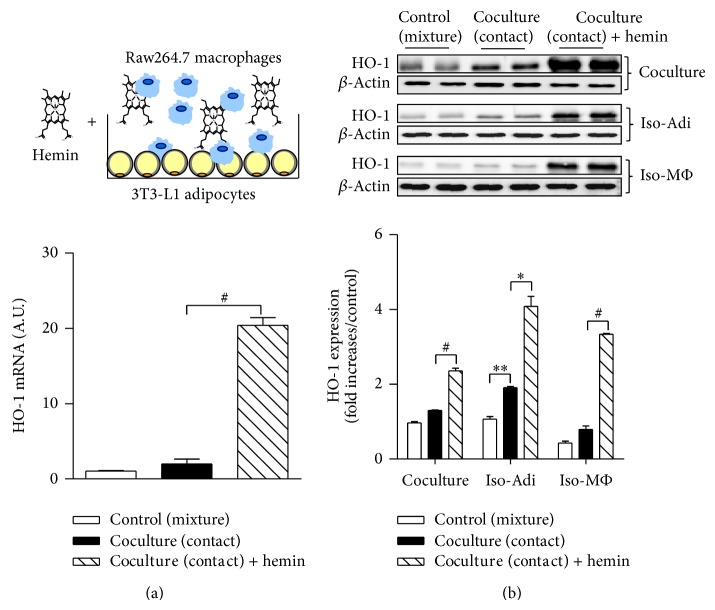
HO-1 induction in adipocyte and macrophage cocultures. Coculture of 3T3-L1 adipocytes and Raw264.7 macrophages were cocultured in direct contact in a ratio 1 : 1 for 24 h in the presence or absence of hemin (10 *μ*M). As a control, adipocytes and macrophages were also cultured separately, with cell numbers per well equal to those in the contact system and mixed after harvesting. (a) Total RNAs were isolated and the level of HO-1 transcripts was analyzed by qRT-PCR. 36B4 was used as a control gene. A.U.: arbitrary units. (b) After 24 h of coculture, the cell types were separated using CD11b microbeads. Levels of HO-1 protein expression were measured in coculture, isolated adipocytes (Iso-Adi), and macrophages (Iso-M*Ф*) by western blotting; *β*-actin was used as a control. The experiment was isolated in duplicate. Results are means ± SEM. ^*^ *P* < 0.05, ^**^ *P* < 0.01, and ^#^ *P* < 0.005.

We first examined the effect of hemin on HO-1 expression in cocultured adipocytes/macrophages, which mimics the inflamed adipose tissue environment in obesity. Pretreatment of adipocytes and macrophages prior to coculture with hemin markedly upregulated HO-1 expression at the transcript and protein levels (Figures 1(a) and 1(b)), and the HO-1 induction was confirmed in the separated adipocytes and/or macrophages retrieved from the coculture (Figure 1(b)).

### 3.2. HO-1 Induction Reduces Release of Inflammatory Cytokines from Cocultured Adipocytes/Macrophages

**Figure 2:**
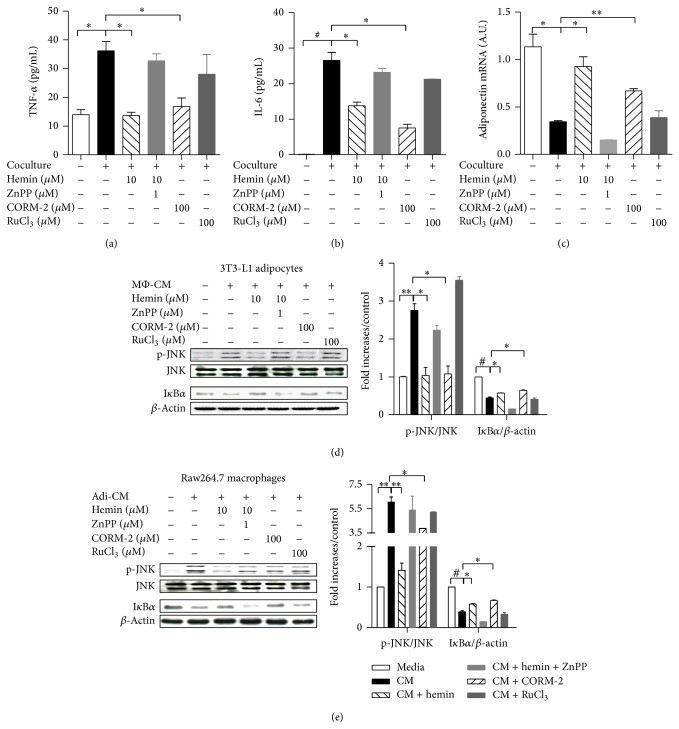
HO-1 induction reduces inflammatory responses in adipocytes and macrophage cocultures. ((a)–(c)) Raw264.7 macrophages were seeded onto 3T3-L1 adipocytes with/without being pretreated with the indicated concentrations of hemin or CORM-2 for 24 h. ZnPP and RuCl_3_ were used as HO-1 competitive inhibitor and CORM-2 negative control, respectively. As a control, adipocytes and macrophages were cultured separately, with cell numbers per well equal to those in the contact system. TNF-*α* (a) and IL-6 (b) protein levels were detected by ELISA. Adiponectin mRNA was detected by qRT-PCR (c). The experiment was set up in triplicate. ((d) and (e)) Adipocytes and macrophages were treated with macrophage-conditioned medium (M*Ф*-CM) and adipocyte-conditioned medium (Adi-CM), respectively, with/without hemin and CORM-2 pretreatment at the indicated concentrations for 1 h. JNK phosphorylation (p-JNK)/JNK and I*κ*B*α* were measured by western blotting. Experiment was performed in duplicate. Results are means ± SEM. ^*^ *P* < 0.05, ^**^ *P* < 0.01, and ^#^ *P* < 0.005.

Next, we examined whether HO-1 induction by hemin affects the production of inflammatory cytokines by the cocultured cells. As shown in Figure 2, HO-1 induction markedly decreased release of the proinflammatory cytokines TNF-*α* (Figure 2(a)) and IL-6 (Figure 2(b)) from the cocultures, while transcript levels of adiponectin increased (Figure 2(c)). The effects of hemin were blunted by ZnPP, a competitive inhibitor of HO-1 (Figures 2(a)–2(c)). Moreover, the CO-releasing molecule, CORM-2, a reagent that mimics the biological effects of HO-1 [13, 16], also attenuated the coculture-induced inflammatory cytokine production (Figures 2(a)–2(c)), indicating that the hemin effect is associated with CO release. The effect of HO-1 induction by hemin appeared to be largely dependent on CO release, because RuCl_3_, which does not liberate CO [13], did not have any effect on the release of inflammatory cytokines.

### 3.3. HO-1 Induction Suppresses Inflammatory Signaling in Cocultured Adipocytes/Macrophages

We further examined the effect of hemin on inflammatory signaling molecules. We treated 3T3-L1 adipocytes or Raw264.7 macrophages with macrophage- or adipocyte-conditioned medium (M*Ф*-CM or Adi-CM) to activate inflammatory signaling pathways. We found that both conditioned media reduced HO-1 expression in adipocytes and macrophages (data not shown), accompanied with increase in phosphorylation of JNK and I*κ*B*α* degradation (Figures 2(d) and 2(e)). Hemin-induced HO-1 expression suppressed the phosphorylation of JNK, as did CORM-2, in both adipocytes (Figure 2(d)) and macrophages (Figure 2(e)). In addition to this, both agents suppressed the increased I*κ*B*α* degradation in M*Ф*/Adi-CM-treated adipocytes/macrophages. ZnPP and RuCl_3_ had no effect.

### 3.4. HO-1 Induction Promotes M2 Macrophage Polarization in Cocultured Adipocytes/Macrophages

**Figure 3:**
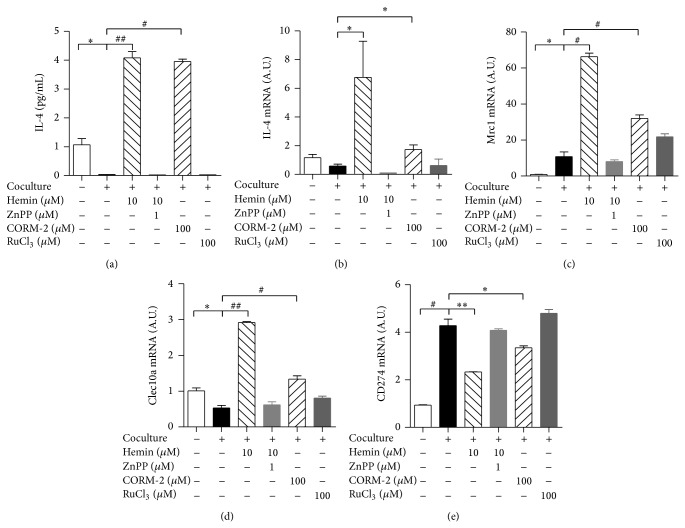
HO-1 induction alters macrophage polarization in cocultures. Raw264.7 macrophages were seed onto 3T3-L1 adipocytes with/without being pretreated with the indicated concentrations of hemin or CORM-2 for 12 h. (a) IL-4 levels in hemin/CORM-2-treated cocultures. (b) Total RNAs were isolated and the level of IL-4 was analyzed by qRT-PCR. Transcript levels of the M2 markers Mrc1 (c) and Clec10a (d) and the M1 marker (CD274) (e) were detected by qRT-PCR; *β*-actin was used as control gene. The experiment was performed in duplicate. Results are means ± SEM. ^*^ *P* < 0.05, ^**^ *P* < 0.01, ^#^ *P* < 0.005, and ^##^ *P* < 0.001. A.U.: arbitrary units.

In order to test whether HO-1 induction affects macrophage polarization in cocultured adipocytes/macrophages, we examined its effect on the expression of M1 and M2 macrophage markers. As shown in Figure 3, HO-1 induction by hemin increased IL-4 release from the cocultures, as did CORM-2 (Figure 3(a)). It also enhanced IL-4 transcript levels (Figure 3(b)). Consistent with this, it also enhanced transcript levels of M2 macrophages markers such as Mrc1 (Figure 3(c)) and Clec10a (Figure 3(d)), while it reduced transcript levels of the M1 macrophage marker CD274 (Figure 3(e)).

### 3.5. HO-1 Induction Reduces HFD-Induced Adipose Tissue Inflammatory Responses

**Figure 4:**
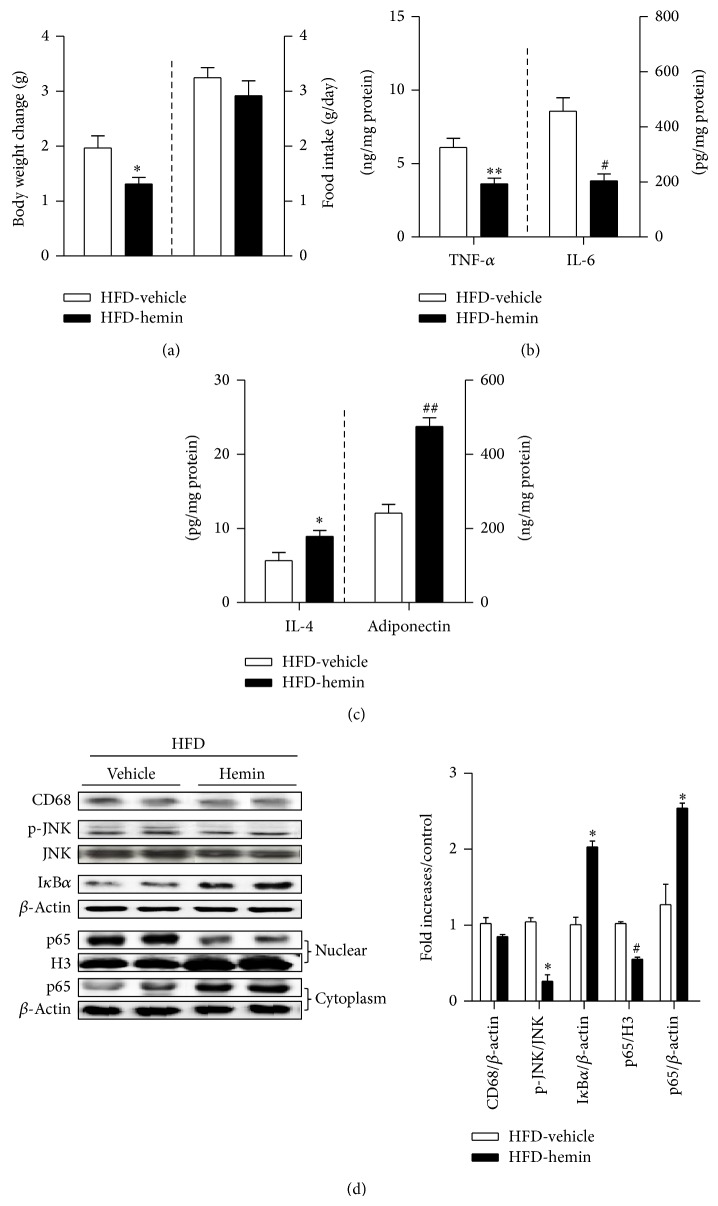
HO-1 induction reduces HFD-induced adipose tissue inflammatory responses. C57BL/6 mice were fed an HFD diet for 2 weeks with hemin injection 3 times per week (*n* = 5). (a) The body weight change and food intake were measured. (b) TNF-*α*, IL-6 levels, and (c) IL-4 and adiponectin levels were measured by ELISA. (d) Expression of a macrophage marker (CD68) and inflammatory molecules (p-JNK/JNK, I*κ*B*α*, and NF-*κ*B p65) in adipose tissue were detected by western blotting. Results are means ± SEM. ^*^ *P* < 0.05, ^**^ *P* < 0.01, ^#^ *P* < 0.005, and ^##^ *P* < 0.001 (compared with HFD-vehicle control).

To extend the* in vitro* findings, we placed C57BL/6 mice on a 60% HFD or control diet for 2 weeks with hemin injected 3 times per week. The body weights of the hemin-treated mice given 60% HFD increased significantly less than those of the vehicle-injected mice, and food intake did not differ between the two groups (Figure 4(a)). As shown in Figure 4, hemin decreased levels of inflammatory cytokines (TNF-*α* and IL-6) in the adipose tissue of HFD-fed mice (Figure 4(b)) and increased IL-4 and adiponectin levels (Figure 4(c)). Hemin treatment did not affect expression of CD68, a macrophage marker (Figure 4(d)). It suppressed phosphorylation of JNK and I*κ*B*α* degradation (Figure 4(d)). We further measured the expression of NF-*κ*B p65, a subunit of the NF-*κ*B transcription complex, in the cytoplasm of adipose tissue and the nuclear fraction. We found that hemin reduced nuclear translocation of p65, pointing to a decrease in NF-*κ*B activation in the adipose tissue of HFD-fed mice (Figure 4(d)). It indicates that HO-1 induction by hemin inhibits inflammatory signaling in adipose tissue, in agreement with the* in vitro* observations.

### 3.6. HO-1 Induction Induces M2 Macrophage Polarization in Adipose Tissue

**Figure 5:**
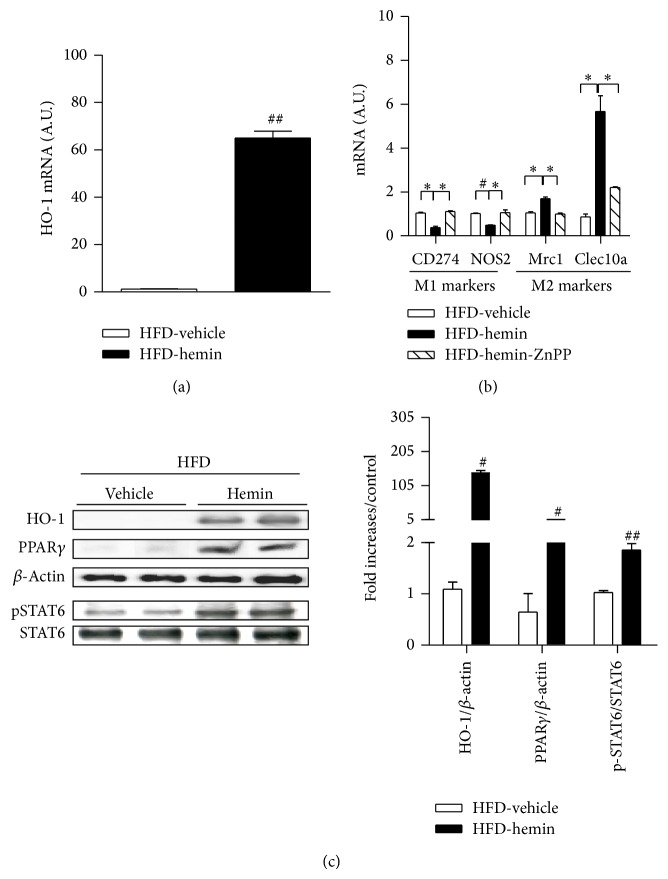
HO-1 induction induces M2 macrophage polarization in adipose tissue. C57BL/6 mice were fed an HFD diet for 2 weeks with hemin alone or hemin in combination with ZnPP injection for 3 times per week (*n* = 5). (a) The level of HO-1 transcripts in adipose tissue was detected by qRT-PCR. (b) Total RNAs were isolated and the levels of M1 marker (CD274, NOS2) and M2 markers (Mrc1, Clec10a) were analyzed by qRT-PCR; *β*-actin was used as control gene. A.U.: arbitrary units. (c) Levels of HO-1, PPAR*γ*, and pSTAT6/STAT6 were detected by western blotting. Results are means ± SEM. ^*^ *P* < 0.05, ^#^ *P* < 0.005, and ^##^ *P* < 0.001 (compared with HFD-vehicle control).

We further examined the effect of HO-1 induction with hemin on macrophage polarization in the adipose tissue of HFD-fed mice. As shown in Figure 5, hemin treatment increased levels of HO-1 transcripts (Figure 5(a)) and protein (Figure 5(c)) in the adipose tissue and also transcripts of M2 marker genes (Mrc1 and Clec10a) (Figure 5(b)) while it decreased M1 marker expression (CD274 and NOS2) (Figure 5(b)). Moreover, the HO-1 inhibitor ZnPP completely blocked the upregulation of M2 markers (Mrc1 and Clec10a) and downregulation of M1 markers (CD274 and NOS2) transcripts (Figure 5(b)). These changes were accompanied by upregulation of PPAR*γ* expression, which has a role in M2 macrophage phenotype switching, and activation of STAT6, a signaling molecule in the IL-4 pathway (Figure 5(c)).

## 4. Discussion

Obesity-induced adipose inflammation is characterized by recruitment of macrophages into adipose tissue and activation of the cells to release inflammatory mediators. Direct cell-cell cross talk between adipocytes and macrophages is likely crucial for promoting inflammatory responses in obese adipose tissue [15, 17]. Using contact cocultured adipocytes/macrophages system and mimicking the inflamed adipose tissue environment in obesity, we first found that the HO-1 induction by hemin significantly reduced levels of inflammatory cytokine (TNF-*α* and IL-6) release from the cocultures. A similar effect was produced by CORM-2, a CO-releasing agent. Induction of HO-1 was noticeable in both of the constituent cell types retrieved from the cocultures, and the effect of hemin was completely blunted by the HO-1 inhibitor ZnPP. These observations indicate that induction by hemin of HO-1, which releases CO via its enzyme activity, is responsible for the reduction of inflammatory cytokine release by both cell types.

Next, we examined the effect of hemin on inflammatory signaling. When adipocytes and macrophages were stimulated with the corresponding conditioned medium, we found that hemin suppressed the activation of the inflammatory signaling molecules JNK and NF-*κ*B in both of the adipocytes and macrophages, while the HO-1 inhibitor ZnPP blunted the hemin-induced suppression of inflammatory signaling molecules in cocultures, indicating that the reduction of inflammatory cytokine release by hemin is due to the inhibitory effect of inflammatory signaling. More importantly, we observed that the CO producer CORM-2 also suppressed phosphorylation of JNK and NF-*κ*B activation. Given that CO, a by-product of heme catabolism by HO-1, exerts potent anti-inflammatory effects by inhibiting JNK/AP-1 binding [18] and/or NF-*κ*B binding [19], the inhibitory effect of hemin on the activation of the inflammatory signaling molecules may be, at least in part, associated with CO production by HO-1, leading to reduced inflammatory cytokines. Because anti-inflammatory M2 macrophages inhibit M1 macrophage-mediated inflammatory responses through inhibition of JNK and NF-*κ*B [20–22], we further inquired whether the suppressed inflammatory signaling by hemin in the cocultured adipocytes/macrophages was associated with macrophage polarization. We found indeed that hemin upregulated transcripts of M2 macrophage markers (Mrc-1, Clec10a, and IL-4), while it decreased M1 macrophage markers (CD274 and TNF-*α*) in the cocultures. These findings indicate that the inhibitory action of hemin on inflammatory signaling leads to switching from the M1 to the M2 macrophage phenotype in the cocultures.

Subsequently, we confirmed the effect of hemin* in vivo* by injecting hemin into HFD-fed mice. Hemin injection markedly upregulated HO-1 expression at the transcript and protein levels, and it reduced levels of the inflammatory cytokines in adipose tissue, and this was also accompanied by reduced activation of inflammatory signaling molecules and increased expression of M2 macrophage markers. In addition, since hemin treatment did not alter CD68 expression in the adipose tissue of the HFD-fed mice but increased M2 marker expression, the anti-inflammatory effect of hemin in adipose tissue may depend on polarization to the M2 phenotype. Consistent with our findings, other studies have reported that the HO-1 system reduces various metabolic complications such as diabetic pathologies and vascular diseases: adipocyte-specific overexpression of HO-1 attenuated HFD-mediated adiposity and vascular dysfunction, increased insulin sensitivity, and improved adipocyte function by increasing adiponectin and by decreasing inflammatory cytokines including MCP-1 [23]. Furthermore, hemin selectively stimulated macrophage polarization towards the anti-inflammatory M2-phenotype in diabetic and/or spontaneously hypertension rats [23–27]. These together with our findings suggest that HO-1 induction by hemin reduces obesity-induced adipose tissue inflammation by promoting macrophages phenotype switching, and this may protect against obesity-related metabolic complications. However, HO-1 induction could be disadvantageous in certain conditions: it can aggravate infection with enterohemorrhagic* Escherichia coli *by reducing nitric oxide production in human enterocytes [28], and it also enhances pancreatic tumor growth and metastasis by increasing angiogenesis [29] and exacerbates intracellular oxidative stress in astroglia, leading to brain injury [30]. Hence, caution is needed if HO-1 inducers are used as a therapeutic target.

It should be noted that PPAR*γ* activation primes monocytes into an enhanced M2 phenotype or has more pronounced anti-inflammatory effects on M1 macrophages [31]. One well-established pathway via which PPAR*γ* controls the inflammatory response is by interfering with inflammatory signaling pathways involving AP-1, NF-*κ*B in activated M1 macrophages [32]. Moreover, it also directly controls the expression of genes involved in inducing the M2 macrophage phenotype, such as the arginase I gene [33]. Because HO-1 enhances the expression and activity of PPAR*γ* [34] and conversely is a target gene for PPAR*γ* signaling [35], the upregulation of PPAR*γ* may promote the polarization towards the M2 phenotype. Moreover, we found that adiponectin, another molecule promoting M2 macrophage polarization [36, 37] and a PPAR*γ* target gene, increased in the adipose tissue of hemin-injected HFD-fed mice, which is consistent with a previous study [23]. HO-1 induction by hemin also increased IL-4 and phosphorylation of STAT6, a typical sign of IL-4 receptor activation, in adipose tissue of HFD-fed mice. Since IL-4 signaling through STAT6 phosphorylation induces transcription of PPAR*γ* and their coactivator, amplifying the expression of signature M2 proteins [38], the increased IL-4/STAT6/PPAR*γ* signaling may be important for hemin-induced M2 macrophage polarization.

In conclusion, we have shown that HO-1 induction by hemin reduces inflammatory responses in cocultured adipocytes/macrophages and in the adipose tissue of HFD-fed mice. The protective effect of HO-1 induction against adipose inflammation was associated with polarization towards the M2 macrophage phenotype via the PPAR*γ* and STAT6 pathway. HO-1 inducing factors such as hemin may be useful for protecting against obesity-induced adipose inflammation.
